# Flavonoids, Phenolics, and Antioxidant Capacity in the Flower of *Eriobotrya japonica* Lindl.

**DOI:** 10.3390/ijms12052935

**Published:** 2011-05-04

**Authors:** Chunhua Zhou, Chongde Sun, Kunsong Chen, Xian Li

**Affiliations:** 1 College of Horticulture and Plant Protection, Yangzhou University, Yangzhou 225009, China; E-Mail: chzhou@yzu.edu.cn; 2 Laboratory of Fruit Quality Biology, The State Agriculture Ministry Laboratory of Horticultural Plant Growth, Development and Quality Improvement, Zhejiang University, Zijingang Campus, Hangzhou 310058, China; E-Mails: adesun2006@zju.edu.cn (C.S.); akun@zju.edu.cn (K.C.)

**Keywords:** *Eriobotrya japonica*, flower, flavonoids, phenolics, antioxidant capacity

## Abstract

Flavonoids and phenolics are abundant in loquat flowers. Methanol had the highest extraction efficiency among five solvents, followed by ethanol. Considering the safety and residue, ethanol is better as extraction solvent. The average content of flavonoids and phenolics of loquat flower of five cultivars were 1.59 ± 0.24 and 7.86 ± 0.87 mg/g DW, respectively, when using ethanol as extraction solvent. The contents of both bioactive components in flowers at different developmental stages and in the various flower tissues clearly differed, with the highest flavonoids and phenolics content in flowers of stage 3 (flower fully open) and petal, respectively. The antioxidant capacity was measured using FRAP, DPPH, and ABTS methods. The values of ABTS method was highest, followed by DPPH, the lowest was FRAP, when using vitamin C equivalent antioxidant capacity (VCEAC) as unit. Correlation analysis showed that the ABTS method showed the highest correlation coefficients with flavonoids and phenolics, *i.e.*, 0.886 and 0.973, respectively.

## 1. Introduction

Free radical (or reactive oxygen species) is an intermediate of aerobic metabolism, and the balance between its generation and removal determines whether the human body would be subject to oxidative destruction. The damage of macromolecules such as nucleic acids, proteins, and membrane lipid, caused by the increased level of free radicals in cells, will trigger a series of aging-related problems [1,2]. Therefore, it has been a central issue to look for free radical scavengers or antioxidants with high efficiency in the anti-aging research. Flavonoids and polyphenols exist widely in plants, and are considered important dietary antioxidants, which play very important roles in the prevention of human oxidative damage [3–6].

The records of many flowers used as Traditional Chinese Medicine (TCM) can be found in ancient books such as “Ben Cao Gang Mu” and “Yang Sheng Lu” *et al*. Research on bioactive components of flowers, healthy food and beverage exploitations of the flowers as raw materials, has received much attention in recent years. However, examination of the antioxidant capacity of flowers was mainly focused on annuals flowers [7–10], while few studies were conducted on the flowers of woody fruit trees [11,12].

Loquat (*Eriobotrya japonica* Lindl.) is a perennial subtropical fruit tree. The fruits can be consumed fresh or processed into jam, juice, wine, syrup, or candied fruits. The flowers (inflorescences) and leaves have been widely used as TCM for treatment of colds, cough, sputum and so on [13]. Many studies demonstrated that large amounts of flavonoids and phenolics were found in the fruit and leaf of loquat [14–20], and both the methanol extract of loquat leaf and its individual fraction exhibited strong antioxidant capacity [18]. At present, reports of identification and analysis of bioactive components in loquat flowers are mainly focused on triterpenoids and amygdalin [21,22], but there is no research on flavonoids, phenolics and antioxidant capacity of loquat flowers. In this study, different methods of antioxidant capacity determination were employed to evaluate the efficiency of scavenging free radicals; simultaneously, the relationship between antioxidant capacity and the contents of flavonoids and phenolics were discussed with the aim to provide scientific basis for the research and exploitation of loquat flower resources.

## 2. Results and Discussion

### 2.1. The Effects of Extract Solvents on Flavonoids, Phenolics and Antioxidant Capacity

The extraction efficiency of flavonoids and total phenolics in loquat flowers varied considerably between different organic solvents, and the antioxidant capacities of loquat flowers also differed (Table 1). Antioxidant capacities measured by three different methods were in the following order: ABTS^+^ assay > DPPH assay > FRAP assay. Thaipong *et al*. [23] measured the antioxidant capacity in methanol and dichloromethane extracts of guava fruit by the above three methods, and also found that the value determined by ABTS^+^ assay was highest, but those determined by DPPH and FRAP assay were similar to each other, though the results using DPPH was slightly higher than that of FRAP. The flavonoids, phenolics and antioxidant capacity of loquat flowers were lower than those of *Stachys lavandulifolium* flowers, but higher than those of *Alcea kardica* flowers [24]. Although methanol had the highest extraction efficiency, ethanol was used as extraction solvent in the following experiment due to it providing higher safety and lowed residue than other solvents [25].

### 2.2. The Effects of Cultivars on Flavonoids, Phenolics and Antioxidant Capacity

Bioactive components and antioxidant capacities may be significantly different among different cultivars [26–29]. Although our previous study showed that the contents of oleanolic acid, ursolic acid and amygdalin in the loquat flower did not vary greatly among different cultivars [22], some differences existed in the contents of flavonoids and phenolics in the loquat flowers of five cultivars tested in the present study, where the highest contents were found in “Dayeyangdun” and the lowest in “Jiajiao”. The average content of flavonoids and phenolics of these five cultivars were 1.59 ± 0.24 and 7.86 ± 0.87 mg/g DW, respectively. Accordingly, differences were also found in the antioxidant capacities of loquat flower ethanol extracts from five cultivars determined by FRAP, DPPH and ABTS^+^ assays, where the highest antioxidant capacity was also found in “Dayeyangdun” and the lowest in “Jiajiao” (Table 2).

### 2.3. The Effects of Developmental Stages on Flavonoids, Phenolics and Antioxidant Capacity

**Figure 1.**
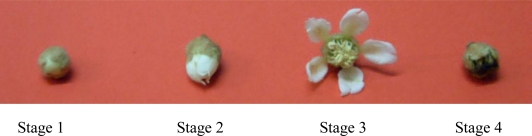
Different development stages of loquat flowers. The four developmental stages of loquat flowers were divided according to the flower opening degree (*i.e.*, stage 1, flower bud; stage 2, the flower is partially open; stage 3, the flower is fully open; stage 4, the petals of the flower have fallen off).

The composition of bioactive components and antioxidant capacity in the plant were significantly affected by the developmental stages [30–32]. The effects of developmental stages (Figure 1) on the flavonoids, phenolics contents and antioxidant capacity in loquat flower were similar to that on amygdalin content [22]. Flower at stage 3 contained the highest flavonoid and phenolic content (3.01 ± 0.13 and 13.53 ± 0.38 mg/g DW, respectively) and showed strongest antioxidant capacity with different assays. Flavonoid and phenolic content and antioxidant capacity of flowers at stage 3 were significantly different to those at the other three stages, and were followed by flowers at stage 2. Flowers at stage 1 and stage 4 contained lower flavonoid and phenolic content, and had lower antioxidant capacities (Table 3). Therefore, it is suggested that the best harvesting time for loquat flowers may be between stage 2 and stage 3 for utilization of its flavonoids and phenolics.

### 2.4. The Flavonoids, Phenolics and Antioxidant Capacity in the Various Flower Tissues

The bioactive components and antioxidant capacity in different tissues of the same plant organs vary greatly [30,33,34]. Results showed that petals contained the highest content of flavonoids and phenolics (7.45 ± 0.38 and 19.63 ± 2.72 mg/g DW, respectively), and had highest antioxidant capacities with values of 4.24 ± 0.04, 6.73 ± 0.04 and 7.19 ± 0.17 VCEAC mg/g DW as determined by the FRAP, DPPH and ABTS^+^ assays, respectively. Flavonoids and phenolics contents and antioxidant capacity of petal were significantly different from those of the other three flower tissues, followed by pistil plus stamen. Peduncle and sepal contained lower flavonoid and phenolic content, and had weaker antioxidant capacities (Table 4). These results can explain why the flower at stage 3 had the highest flavonoid and phenolic content and strongest antioxidant capacities.

### 2.5. Correlation Analysis

Many studies showed that the contents of flavonoids and phenolics were positively correlated to the antioxidant capacities in plant extracts [35–37]. Similar results were also obtained in this research (Table 5); correlations between flavonoid and phenolic content and antioxidant capacities obtained from three different assays were positively high (0.694 ≤ *r*^2^ ≤ 0.785; 0.762 ≤ *r*^2^ ≤ 0.947), especially among flavonoids, phenolics contents and antioxidant capacities based on ABTS^+^ assay (*r*^2^ = 0.785**; *r*^2^ = 0.947**). The lowest correlation was found between flavonoid and phenolic content and antioxidant capacities obtained by FRAP assay (*r*^2^ = 0.694**; *r*^2^ = 0.762**). Correlations between phenolics content and antioxidant capacities determined by three different methods were higher than those between flavonoids content and antioxidant capacities. Similar results have also been reported previously [35].

**Figure 2.**
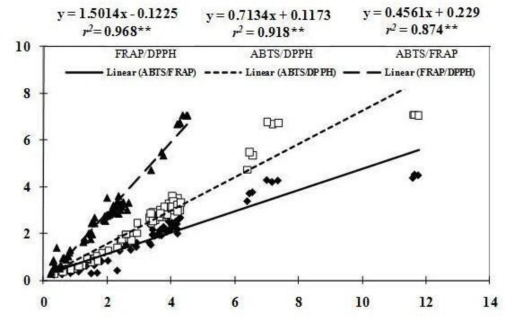
The correlation between VCEAC and different antioxidant capacity detection methods.

As shown in Figure 2, positive correlation (0.874 ≤ *r*^2^ ≤ 0.968) was found among antioxidant capacities determined by FRAP, DPPH and ABTS^+^ methods. The highest correlation (*r*^2^ = 0.968**) existed between antioxidant capacities obtained by FRAP and DPPH assays, while the lowest value (*r*^2^ = 0.874**) was found between antioxidant capacities obtained by FRAP and ABTS^+^ methods. The high relationship between antioxidant capacities measured by these three different methods was also investigated in other crops. Awika *et al*. [38] observed that antioxidant capacities in sorghums and their products determined by ABTS^+^ and DPPH assays were highly correlated. In the study of guava, Thaipong *et al*. [23] also found linear correlations between antioxidant capacities obtained from FRAP, DPPH, ABTS^+^ and ORAC (oxygen radical absorbance capacity) assays, with the highest correlation between FRAP and ABTS assays, and lowest between DPPH and ORAC assays.

## 3. Experimental Section

### 3.1. Plant Materials

Loquat flowers of five cultivars, *i.e.*, Ruantiaobaisha, Dahongpao, Jiajiao, Baozhu, and Dayeyangdun, were collected in winter from the Yuhang District, Hangzhou, Zhejiang Province, China. Flowers at the four developmental stages (*i.e.*, stage 1, stage 2, stage 3, stage 4) and four tissues (*i.e.*, petal, sepal, pistil plus stamen, peduncle) of Ruantioabaisha cultivar were used to study the effect of different developmental stages and flower tissues on flavonoid and phenolic content and antioxidant capacities. All the plant materials were dried in a microwave oven (Panasonic) because of its high efficiency and low cost until the weight was unchangeable, and then ground into powder and stored at −20 °C until analysis.

### 3.2. Extraction of Flavonoids and Phenolics

Plant powder of 0.5 g was solubilized in 20 mL anhydrous ethanol and other organic solvents (AR) purchased from Eage Chemical Reagent Co. Ltd. (Shanghai) for 2 h followed by 30 min ultrasonic extraction by TBT/C-YCL 500Tt/3P (D) ultrasonic machine (Sinobest electronic Co. Ltd., Jining, Shangdong Province, China) under the selected frequency and power (47 kHz and 500 W) combination. The samples were extracted twice and both extracts were combined and evaporated to dryness at 35 °C The residue was dissolved in 1 mL methanol and transferred to an Eppendorf tube, and then centrifuged at 8000 × g for 10 min. After centrifugation, the supernatant was diluted with ethanol for the determination of flavonoids, total phenolic content and antioxidant capacity.

### 3.3. Determination of Flavonoid and Phenolic Content

Total flavonoids were determined by the procedure of Zhou *et al*. [39] with some modification. Aliquots (1 mL) of loquat flower extracts were placed in two test tubes, respectively. 7 mL methanol was added to one tube. In the other tube, 1 mL 2% ZrOCl_2_·8H_2_O and 6 mL methanol was added. The solution was mixed again and placed into water bath at 30 °C for 1 h. The absorbance was measured at 420 nm with DU-8000 UV-Vis spectrophotometer (Beckman Coulter, USA) and ΔOD was calculated. The amount of total flavonoids was calculated as a rutin equivalent from the standard curve, and expressed as mg rutin/g dry plant material (mg/g DW). All measurements were repeated three times.

Total phenolics were determined by Folin-Ciocalteu procedure [40]. Aliquots (0.5 mL) of loquat flower extracts were transferred into the test tubes and their volumes were made up to 4.5 mL with distilled water. After addition of 0.5 mL Folin-Ciocalteu reagent and 1 mL 7% aqueous sodium carbonate solution, tubes were vortexed and placed into water bath at 30 °C for 1 h. Then absorbance of mixtures was recorded at 760 nm with DU-8000 UV-Vis spectrophotometer (Beckman Coulter, USA) against a blank containing 0.5 mL of extraction solvent. The amount of total phenolics was calculated as a chlorogenic acid equivalent from the standard curve, and expressed as mg chlorogenic acid/g dry plant material (mg/g DW). All measurements were done in triplicate.

### 3.4. Determination of Antioxidant Capacity

The FRAP (ferric reducing antioxidant power) assay was carried out by the methods of Deighton *et al*. [41] and Kim *et al*. [42] with some modification. The FRAP reagent was freshly prepared by mixing 25 mL of 300 mmol/L sodium acetate buffer (pH 3.6), 2.5 mL of 10 mmol/L TPTZ (2,4,6-tripyridyl-*s*-triazine) solution, and 2.5 mL of 20 mmol/L ferric chloride solution. The absorbance at 595 nm was measured 10 min after the mixing of 100 μL of extract (or antioxidant standard) with 900 μL of FRAP reagent, using a spectrophotometer (Beckman Coulter, USA), ethanol was used as control and Vc (Vitamin C) as antioxidant standard. The antioxidant capacity was expressed as Vitamin C equivalent antioxidant capacity (VCEAC, mg/g DW). All determinations were repeated three times.

The DPPH (2,2-diphenyl-1-picrylhydrazyl) assay was measured according to the procedures of Bao *et al*. [43] and Kim *et al*. [42] with some modification. The absorbance at 515 nm was measured 6 h after the mixing of 100 μL of extract (or antioxidant standard) with 3.9 mL DPPH (60 μmol/L) solution, using a spectrophotometer (Beckman Coulter, USA), ethanol used as control and Vc as antioxidant standard. The antioxidant capacity was expressed as VCEAC (mg/g DW). All measurements were done in triplicate.

The ABTS [2,2′-azinobis(3-ethylbenzothiazoline-6-sulfonic acid)] assay was measured by the procedures of Delgado-Andrade *et al*. [44] and Kim *et al*. [42] with some modification. ABTS^+^ was produced by reaction of 7 mmol/L ABTS stock solution with 2.45 mmol/L potassium persulfate and allowing the mixture to stand in the dark at room temperature for 12–16 h before use. The ABTS^+^ solution (stable for 2 day) was diluted with 5 mmol/L phosphate-buffered saline (pH 7.4) to an absorbance of 0.70 ± 0.02 at 730 nm. The absorbance at 730 nm was investigated 10 min after the mixing of 100 μL of fruit extract (or antioxidant standard) with 3.9 mL ABTS^+^ solution, using a spectrophotometer (Beckman Coulter, USA), ethanol used as control and Vc as antioxidant standard. The antioxidant capacity was expressed as VCEAC (mg/g DW). All measurements were repeated in triplicate.

### 3.5. Statistical Analysis

Standard deviations (SD) were calculated by Origin (Microcal Software Inc., Northampton, MA, USA). Duncan’s new multiple range method test (DPS version 3.11) was calculated for mean separation. Differences were considered significant at *P* < 0.05.

## 4. Conclusions

Loquat flowers are rich in flavonoids and phenolics, and the methanol and ethanol extracts of loquat flowers showed high antioxidant capacities obtained by FRAP, DPPH and ABTS^+^ assays. The antioxidant capacity determined by ABTS^+^ assay was highest among these three methods when using VCEAC as unit, and highest correlation coefficients was determined between VCEAC when obtained by ABTS^+^ assay and flavonoids as well as phenolics. Therefore, the ABTS^+^ assay may be the best method for antioxidant capacity determination in loquat flower extracts. These results may provide a theoretical basis for further exploitation of loquat flower resources.

## Figures and Tables

**Table 1.** Flavonoids, phenolics and antioxidant capacity in extracts prepared by different solvents.

**Extract Solvent**	**Component (mg/g DW)**		**Antioxidant Capacity (VCEAC mg/g DW)**		

	**Flavonoids**	**Phenolics**	**FRAP**	**DPPH**	**ABTS**
**Methanol**	6.36 ± 0.41 *^a^*	36.80 ± 4.28 *^a^*	4.46 ± 0.08 *^a^*	7.07 ± 0.01 *^a^*	11.67 ± 0.09 *^a^*
**Ethanol**	1.59 ± 0.04 *^b^*	8.33 ± 0.20 *^b^*	2.32 ± 0.28 *^b^*	3.31 ± 0.20 *^b^*	4.06 ± 0.14 *^b^*
**Acetone**	1.00 ± 0.02 *^c^*	2.88 ± 0.20 *^c^*	0.80 ± 0.03 *^c^*	1.15 ± 0.14 *^c^*	1.84 ± 0.18 *^c^*
***n*-Butyl alcohol**	0.80 ± 0.06 *^c^*	2.76 ± 0.20 *^c^*	0.65 ± 0.05 *^c^*^,^*^d^*	0.91 ± 0.04 *^c^*^,^*^d^*^,^*^e^*	1.45 ± 0.14 *^c^*^,^*^d^*
**Ethyl acetate**	0.72 ± 0.04 *^c^*	1.34 ± 0.44 *^c^*	0.42 ± 0.10 *^d^*	0.55 ± 0.07 *^d^*^,^*^e^*^,^*^f^*	1.01 ± 0.20 *^d^*

The materials used in this section were “Ruantaibaisha” at stage 2 (flower partially open); The order of the extract solvent is according to the polarity magnitude; VCEAC means Vitamin C equivalent antioxidant capacity; Different letters within each column indicate significant differences (*P* < 0.05).

**Table 2.** Flavonoids, phenolics and antioxidant capacity in flowers of different cultivars.

**Cultivar**	**Component (mg/g DW)**		**Antioxidant Capacity (VCEAC mg/g DW)**		
	**Flavonoids**	**Phenolics**	**FRAP**	**DPPH**	**ABTS**
**Baozhu**	1.35 ± 0.01 *^b^*	7.39 ± 0.10*^c^*	2.02 ± 0.10 *^c^*^,^*^d^*	2.80 ± 0.06 *^b^*^,^*^c^*	3.75 ± 0.11 *^b^*
**Dahongpao**	1.79 ± 0.10 *^a^*	7.77 ± 0.35 *^b^*^,^*^c^*	2.28 ± 0.07 *^b^*	2.89 ± 0.05 *^b^*^,^*^c^*	3.64 ± 0.24 *^b^*
**Dayeyangdun**	1.81 ± 0.07 *^a^*	9.15 ± 0.20 *^a^*	2.60 ± 0.09 *^a^*	3.22 ± 0.19 *^a^*	4.25 ± 0.08 *^a^*
**Jiajiao**	1.30 ± 0.01 *^b^*	6.73 ± 0.21 *^d^*	1.94 ± 0.06 *^d^*	2.62 ± 0.12 *^c^*	3.52 ± 0.10 *^b^*
**Ruantiaobaisha**	1.70 ± 0.06 *^a^*	8.24 ± 0.28 *^b^*	2.18 ± 0.06 *^b^*^,^*^c^*	3.03 ± 0.11 *^a^*^,^*^b^*	3.63 ± 0.18 *^b^*
**Average**	1.59 ± 0.24	7.86 ± 0.87	2.20 ± 0.25	2.91 ± 0.23	3.76 ± 0.29

Materials used in this section were collected at stage 2 (flower partially open); Different letters within each column indicate significant differences (*P* < 0.05).

**Table 3.** Flavonoids, phenolics and antioxidant capacity in flowers at different developmental stages.

**Developmental Stage**	**Component (mg/g DW)**		**Antioxidant Capacity (VCEAC mg/g DW)**		

	**Flavonoids**	**Phenolics**	**FRAP**	**DPPH**	**ABTS**
**Stage 1**	1.16 ± 0.04 *^c^*	8.25 ± 0.07 *^b^*	1.56 ± 0.05 ^c^	2.52 ± 0.14 *^c^*	3.22 ± 0.24 *^c^*
**Stage 2**	1.50 ± 0.05 *^b^*	8.74 ± 0.15 *^b^*	2.35 ± 0.05 *^b^*	3.41 ± 0.18 *^b^*	4.10 ± 0.09 *^b^*
**Stage 3**	3.01 ± 0.13 *^a^*	13.53 ± 0.38 *^a^*	3.61 ± 0.21 *^a^*	5.19 ± 0.41 *^a^*	6.48 ± 0.08 *^a^*
**Stage 4**	1.24 ± 0.04 *^c^*	6.72 ± 0.67 *^c^*	2.10 ± 0.15 *^b^*	3.03 ± 0.27 *^b^*^,^*^c^*	3.88 ± 0.20 *^b^*

Materials used in this section were “Ruantiaobaisha”. Different letters within each column indicate significant differences (*P* < 0.05).

**Table 4.** Flavonoids, phenolics and antioxidant capacity of different flower tissues.

**Flower Tissue**	**Component (mg/g DW)**		**Antioxidant Capacity (VCEAC mg/g DW)**		

	**Flavonoids**	**Phenolics**	**FRAP**	**DPPH**	**ABTS**
**Peduncle**	1.19 ± 0.07 *^b^*	4.88 ± 0.17b *^c^*	1.50 ± 0.02 *^c^*	1.89 ± 0.12 *^c^*	2.54 ± 0.08 *^c^*
**Petal**	7.45 ± 0.38 *^a^*	19.63 ± 2.72 *^a^*	4.24 ± 0.04 *^a^*	6.73 ± 0.04 *^a^*	7.19 ± 0.17 *^a^*
**Pistil + Stamen**	1.81 ± 0.07 *^b^*	7.99 ± 0.25 *^b^*	2.34 ± 0.14 *^b^*	3.06 ± 0.16 *^b^*	4.12 ± 0.09 *^b^*
**Sepal**	1.15 ± 0.14 *^b^*	4.26 ± 0.66 *^c^*	1.31 ± 0.09 *^c^*	1.78 ± 0.19 *^c^*	2.69 ± 0.27 *^c^*

The materials used in this section were “Ruantiaobaisha” at stage 2 (flower partially open) because the flowers in thinned inflorescences are most abundant in this stage. Different letters within each column indicate significant differences (*P* < 0.05).

**Table 5.** Correlation analysis between flavonoids, phenolics and antioxidant capacity.

**Antioxidant Capacity**	**Flavonoids**	**Phenolics**
**FRAP**	*y* = 0.5615*x* + 0.8809 (*r*^2^ = 0.694**)	*y* = 0.1318*x* + 0.8335 (*r*^2^= 0.762**)
**DPPH**	*y* = 0.9107*x* + 1.0784 (*r*^2^ = 0.785**)	*y* = 0.2091*x* + 1.0382 (*r*^2^ = 0.824**)
**ABTS**	*y* = 1.1812*x* + 1.5189 (*r*^2^ = 0.785**)	*y* = 0.301*x* + 1.2275 (*r*^2^ = 0.947**)
